# Clinic presentation delay and tuberculosis treatment outcomes in the Lake Victoria region of East Africa: A multi-site prospective cohort study

**DOI:** 10.1371/journal.pgph.0002259

**Published:** 2023-08-30

**Authors:** Grace E. Mulholland, Michael E. Herce, Brenda A. Okech, Kidola Jeremiah, Ubaldo M. Bahemuka, Zachary A. Kwena, Gertrude Nanyonjo, Janet Seeley, Audrey Pettifor, Michael Emch, Sharon S. Weir, Jessie K. Edwards

**Affiliations:** 1 Department of Epidemiology, University of North Carolina at Chapel Hill, Chapel Hill, North Carolina, United States of America; 2 Institute for Global Health and Infectious Diseases, School of Medicine, University of North Carolina at Chapel Hill, Chapel Hill, North Carolina, United States of America; 3 UVRI-IAVI HIV Vaccine Program Limited, Entebbe, Uganda; 4 Mwanza Research Centre, National Institute for Medical Research, Mwanza, Tanzania; 5 Medical Research Council/Uganda Virus Research Institute & London School of Hygiene and Tropical Medicine, Uganda Research Unit, Entebbe, Uganda; 6 Kenya Medical Research Institute, Nairobi, Kenya; 7 Global Health and Development Department, London School of Hygiene and Tropical Medicine, London, United Kingdom; 8 Department of Geography, University of North Carolina at Chapel Hill, Chapel Hill, North Carolina, United States of America; Zuckerberg San Francisco General Hospital and Trauma Center, UNITED STATES

## Abstract

In the Lake Victoria region of East Africa, little is known about delays between tuberculosis (TB) symptom onset and presentation at a clinic. Associations between clinic presentation delay and TB treatment outcomes are also poorly understood. In 2019, we abstracted data from routine TB treatment records for all adults (n = 776) initiating TB treatment in a 6-month period across 12 health facilities near Lake Victoria. We interviewed 301 cohort members and assessed whether they experienced a clinic presentation delay longer than 6 weeks. We investigated potential clinical and demographic correlates of clinic presentation delay and examined the association between clinic presentation delay and an unfavorable TB treatment outcome (death, loss to follow-up, or treatment failure). Clinic presentation delay was common, occurring among an estimated 54.7% (95% CI: 48.9%, 61.2%) of cohort members, though no specific correlates were identified. Clinic presentation delay was slightly associated with unfavorable TB treatment outcomes. The 180-day risk of an unfavorable outcome was 14.2% (95% CI: 8.0%, 20.4%) among those with clinic presentation delay, compared to 12.7% (95% CI: 5.1%, 20.3%) among those presenting earlier. Multi-level community-based interventions may be necessary to reduce clinic presentation delays in communities near Lake Victoria.

## Introduction

Effective tuberculosis (TB) treatment rapidly reduces infectiousness [1–3], curtailing onward transmission of *M*. *tuberculosis* [4, 5] and substantially reducing the risk of mortality and other poor clinical outcomes [6]. TB remains, however, among the world’s most deadly infectious diseases and a leading cause of death in low- and lower-middle-income countries [7]. Many countries with high TB incidence rely heavily on passive case finding [8, 9]. This is a patient-initiated path to diagnosis which necessitates that a person experiences TB symptoms, determines a need for care, and seeks care at a health facility with TB diagnostic capabilities [10]. This path presents multiple opportunities for delays between the onset of symptoms and presentation at a health facility. Clinic presentation delay may be especially common in hard-to-reach populations with limited access to health care services, such as in fishing communities near the shores of Lake Victoria [11].

Clinic presentation delay may contribute to or predict suboptimal treatment outcomes, yet few studies have explored this relationship. In this study, we describe clinic presentation delay among people who initiated TB treatment at 12 public sector health facilities in the Lake Victoria region of Kenya, Tanzania, and Uganda. We also estimate associations between clinical and demographic characteristics and clinic presentation delay, and we describe reported reasons for clinic presentation delay. Finally, we estimate the association between clinic presentation delay and unfavorable TB treatment outcomes.

## Methods

### Study design

We conducted a longitudinal cohort study with data collection proceeding in three main stages at each of the 12 health facilities included in the study. First, the study population (“cohort”) was enumerated from health facility records, and basic demographic and clinical data were abstracted for members of the cohort. Second, a subset of cohort members (a “subcohort”) was recruited for face-to-face interviews, and clinic presentation delay was ascertained among members of the subcohort. Third, at study closure, available TB treatment outcomes were abstracted for members of the cohort.

### Setting and participants

**Fig 1:**
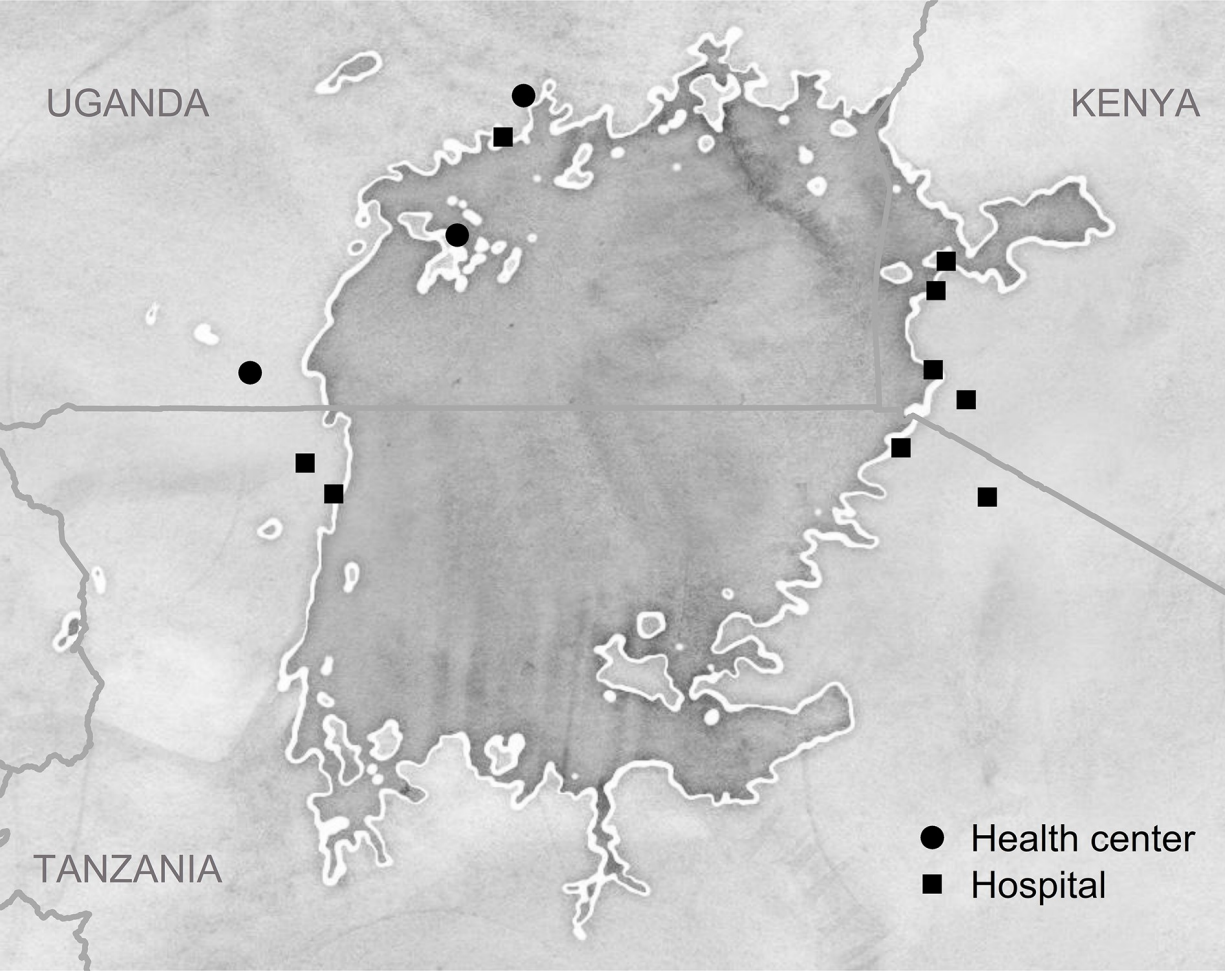
Health facilities where records were abstracted to enumerate the study cohort. The study team abstracted basic clinical and demographic data for a census of people initiating TB treatment over a 6-month period at these 9 health centers and 3 hospitals. A subset of cohort members (a “subcohort”) was recruited from across the 12 facilities for additional data collection via face-to-face interviews. Data were collected in the East Africa TB/HIV and Mobility Study (2019). Map data are from OpenStreetMap (openstreetmap.org/copyright); map tiles are by Stamen Design (http://maps.stamen.com/#watercolor/8/-1.300/32.900).

The study was conducted at 12 public sector health facilities located near the shores of Lake Victoria. Facilities were selected according to the following criteria: high volume of people on TB treatment, availability of or linkage to Xpert MTB/RIF diagnostic testing, capacity to initiate anti-TB treatment for people with drug-susceptible and drug-resistant TB, maintenance of person-level TB treatment records, and willingness to facilitate records access. Facilities were in Kyotera, Kalangala, and Wakiso districts in Uganda; in Suba, Mbita, Karungu, and Macalder sub-counties in Kenya; and in Shirati, Tarime, and Bukoba districts in Tanzania (Fig 1). All people ages 18 and older who initiated TB treatment in the 6 months preceding the start of data collection at the selected health facilities were included in the cohort.

### Stage 1: Cohort enumeration and abstraction of demographic and clinical data for cohort members

The study team visited the 12 selected health facilities and consulted TB treatment registers to enumerate the cohort and abstract data on key variables. At each facility, the study team identified, as cohort members, all people who were 18 years of age or older and who initiated TB treatment within the 6-month period preceding the first date of data collection at the health facility. Because the timing of data collection varied somewhat, across the 12 facilities, TB treatment initiation dates among cohort members included in the study ranged from December 2018 to November 2019.

### Stage 2: Recruitment of a subcohort and ascertainment of clinic presentation delay among members of the subcohort

The next stage of data collection involved interviewing a subset of cohort members (a “subcohort”) to assess clinic presentation delay and additional characteristics not captured in routine health records. We set health facility-specific recruitment targets to ensure that the distribution of subcohort members across the health facilities would reflect that of cohort members and that the subcohort would include an approximately equal number of people with and without TB-associated HIV. The study team coordinated with staff at each health facility to integrate the subcohort recruitment process into routine client intake procedures. When a person on TB treatment presented for care, the health facility staff identified whether the individual met the inclusion criteria for the study cohort. If so, they referred the client to the study team who confirmed cohort membership and administered consent and recruitment procedures. Subcohort recruitment continued consecutively as people presented for care until the facility-specific targets were reached.

In face-to-face interviews, subcohort members reported on characteristics not recorded in health facility records, including the exposure, clinic presentation delay. We defined clinic presentation delay categorically in the interview as waiting more than 6 weeks after TB symptom onset to seek care at a health facility. A six-week threshold has been used elsewhere to characterize a prolonged period between onset of TB symptoms and first consulting a healthcare provider [12]. Clinic presentation delay was assessed retrospectively; all cohort members had initiated TB treatment by the time of the interview. Subcohort members were asked if they experienced clinic presentation delay, and if so, why they did not present to a health facility sooner.

### Stage 3: Abstraction of TB treatment outcomes for cohort members

At study closure, which occurred between 3 and 6 months after enumeration of the cohort, the study team returned to the health facilities to abstract cohort members’ TB treatment outcomes. A phone-based tracing study was conducted to confirm treatment outcomes among subcohort members who either initiated treatment at least 6 months before study closure but had no outcome recorded, or had recorded outcomes of “lost to follow-up” or “not evaluated.” Outcomes were updated to the tracing-confirmed outcome for each of the 75 subcohort members successfully traced.

### Ethical considerations

The protocol was approved by the Institutional Review Board of the University of North Carolina at Chapel Hill; the Uganda Virus Research Institute (UVRI) Research Ethics Committee; the Uganda National Council for Science and Technology and the London School of Hygiene and Tropical Medicine Research Ethics Committee; the Kenya Medical Research Institute (KEMRI) Scientific and Ethics Review Unit; and the Medical Research Coordinating Committee of the National Institute for Medical Research (NIMR), Tanzania. The cohort was enumerated from TB treatment registers under a waiver of informed consent; therefore, the health facility staff redacted sensitive information such as name, patient record number, and contact information from the materials and the study team created the cohort from the redacted records. The study team obtained written informed consent from the cohort members who were recruited for the subcohort survey. Members of the subcohort were compensated for transportation and their time.

### Inclusivity in global research

Additional information regarding the ethical, cultural, and scientific considerations specific to inclusivity in global research is included in the S1 Checklist.

### Statistical analysis

We conducted our analyses using SAS 9.4 [13] and R 4.0.3 [14]. One cohort member with missing HIV status was excluded from all analyses. We used map data from OpenStreetMap, licensed under the Open Data Commons Open Database License (ODbL) by the OpenStreetMap Foundation (OSMF) [15]. Map tiles are by Stamen Design, licensed under CC BY 3.0 [16].

#### Imputation of missing clinic presentation delay and covariate data

Clinic presentation delay and detailed covariate data were missing by design for cohort members who did not participate in the subcohort survey, and a small number of subcohort members (n = 9; 3.0%) had an undefined time to clinic presentation. Missing values were imputed using multiple imputation by fully conditional specification [17]. We included expected predictors of the value or missingness of clinic presentation delay and all variables from the analysis models. The full set of variables included in the imputation process, coding details, and the variable-specific imputation methods used are provided in the S1 Table. Models for each incomplete variable included all other variables in the table. We generated 200 complete data sets, and for all analyses using the multiply imputed data, we used Rubin’s rules [18] to summarize point estimates and standard errors.

#### Predictors of clinic presentation delay

We estimated the prevalence of clinic presentation delay in the cohort in each imputation by fitting an intercept-only log-binomial model. We also fit separate log-binomial models within strata of clinical and demographic characteristics, and we computed prevalence ratios (PRs) to assess the association between clinic presentation delay and these characteristics. Finally, we reported the proportion of subcohort members with clinic presentation delay who experienced delay for various reasons.

#### Estimating risk of an unfavorable TB treatment outcome by clinic presentation delay

We used the Nelson-Aalen estimator [19, 20] to compute the risk of an unfavorable TB treatment outcome within 180 days of treatment initiation. We followed cohort members from their date of TB treatment initiation until the date of an unfavorable treatment outcome, transfer out, outcome recorded as “unknown” or “not evaluated,” study closure (i.e., the date of outcome data collection), or 180 days, whichever occurred first. Unfavorable TB treatment outcome was a composite outcome that included death, loss to follow-up, and treatment failure. We estimated risks up to 180 days to align with the typical 6-month drug-susceptible TB treatment duration. Missing death dates (n = 6) were imputed as the midpoint between the last recorded contact with the health facility and study closure. Missing dates for loss to follow-up (n = 14) were imputed as 60 days beyond the last recorded contact with the health facility, to approximate a treatment interruption of two months [21].

We estimated the 180-day risk of an unfavorable TB treatment outcome overall and within strata defined by HIV status and sex. Then, we calculated risk differences, comparing risks among those with and without clinic presentation delay. We used the delta method [22] to estimate standard errors of the risk differences. We also conducted two sensitivity analyses. In one, we repeated the main analysis among only the subset of cohort members with measured (i.e., non-imputed) values for clinic presentation delay. In a second sensitivity analysis, we estimated the association between clinic presentation delay and the most common unfavorable TB treatment outcome, death.

## Results

Cohort and subcohort characteristics are presented in Table 1. Most (86.1%) of the 775 cohort members had pulmonary TB, over 90% were new TB cases, and half (49.7%) had TB-associated HIV. About 40% of cohort members were female, and the median age was 39 (IQR: 29–50) years. TB was bacteriologically confirmed among 47.4% of the cohort members, and drug-resistant TB was recorded for less than 3%. The subcohort (n = 301, 38.8% of the cohort) was generally similar to the full cohort with respect to the distribution of abstracted variables.

**Table 1:** Characteristics of subcohort and cohort members. Basic demographic and clinical characteristics are presented for all people who initiated TB treatment over a 6-month period (the “cohort”). Additional characteristics are presented for cohort members who participated in face-to-face interviews (the “subcohort”).

	Subcohort (n = 301)		Cohort (n = 775)	
	n	%	n	%
Female	131	43.5	317	40.9
Age group				
18 to 24 years	36	12.0	92	11.9
25 to 34 years	73	24.3	199	25.7
35 to 49 years	124	41.2	276	35.6
50 years or older	68	22.6	208	26.8
Country of treatment initiation				
Kenya	49	16.3	105	13.5
Tanzania	125	41.5	341	44.0
Uganda	127	42.2	329	42.5
TB site				
Pulmonary	262	87.9	659	86.1
Extra-pulmonary	36	12.1	106	13.9
Missing	3		10	
Patient type				
New	265	89.5	706	91.9
Relapse	27	9.1	56	7.3
Treatment after failure	1	0.3	1	0.1
Treatment after loss to follow-up	3	1.0	5	0.7
Other (unspecified)/Missing	5		7	
HIV-associated TB	157	52.2	386	49.8
TB bacteriologically confirmed by GeneXpert, culture, or smear	160	53.2	367	47.4
by GeneXpert	141	46.8	321	41.4
Drug-resistant TB^1^	16	5.3	20	2.6
Resided in area^2^ where health facility is located				
Yes	177	86.3	400	80.0
No	28	13.7	100	20.0
Missing	96		275	
Participated in subcohort interview, providing additional information (including for characteristics shown below)	301	100.0	301	38.8
Marital status				
Married or cohabitating with sexual partner	152	50.5		
Separated/Divorced	71	23.6		
Single (never married)	42	14.0		
Widowed	36	12.0		
Highest level of education completed				
Less than primary school	118	39.2		
Primary school	140	46.5		
Form 6	12	4.0		
College (vocational, tertiary, or non-tertiary) or university	31	10.3		
Employment status				
Formally employed	50	16.6		
Informally employed	157	52.2		
Not employed, seeking work	44	14.6		
Not employed, not seeking work	50	16.6		
Worked in the fishing industry in the past 12 months				
No	247	84.4		
Yes	47	15.6		
Missing	7			
Worked in a mine in the past 12 months				
No	257	89.3		
Yes	32	10.7		
Missing	12			
Any household members went to bed hungry in the past 30 days				
No	209	71.1		
Yes	85	28.9		
Missing	7			

^1^Includes additional diagnoses noted by time of study closure.

^2^Sub-county in Kenya; district in Uganda and Tanzania.

Data were collected at 12 health facilities in the Lake Victoria region of East Africa in the East Africa TB/HIV and Mobility Study (2019).

Clinic presentation delay was reported by 54.1% (n = 158) of subcohort members with a defined time to clinic presentation. We estimated that 54.7% (95% CI: 48.9%, 61.2%) of cohort members experienced clinic presentation delay. Table 2 presents prevalence ratios estimating associations between clinic presentation delay and other characteristics. The prevalence ratios furthest from the null compared prevalence by educational attainment, with, for example, those who completed Form 6 1.19 times (95% CI: 0.88, 1.62) as likely to have experienced this delay as those who had not completed primary school. Clinic presentation delay among people who worked in the fishing industry in the preceding 12 months was 0.87 (95% CI: 0.62, 1.22) times the prevalence among those who did not recently work in this industry, and people with TB-associated HIV were 0.87 (95% CI: 0.70, 1.09) times as likely to have experienced clinic presentation delay as those without HIV. Other associations were closer to the null (1), and all 95% confidence intervals included the null.

**Table 2:** Prevalence of clinic presentation delay by characteristics, and associations between characteristics and delay.

	Prevalence of clinic presentation delay^1^ among subcohort members^2^		Prevalence (%) of clinic presentation delay among cohort members (95% CI)^3^	Prevalence ratio among cohort members (95% CI)
	n	%		
Overall	158	54.1	54.7 (48.9, 61.2)	
Sex				
Male	90	54.9	55.7 (48.4, 64.1)	1
Female	68	53.1	53.2 (44.8, 63.2)	0.95 (0.77, 1.19)
Age group				
18 to 24 years	19	55.9	55.9 (41.5, 75.1)	0.98 (0.71, 1.37)
25 to 34 years	36	51.4	52.1 (41.7, 65.1)	0.92 (0.71, 1.19)
35 to 49 years	68	56.2	56.8 (48.8, 66.0)	1
50 years or older	35	52.2	53.4 (42.8, 66.8)	0.94 (0.73, 1.22)
Country of treatment initiation				
Kenya	26	53.1	52.7 (40.6, 68.4)	0.95 (0.70, 1.27)
Tanzania	64	52.5	54.2 (45.9, 63.9)	0.97 (0.77, 1.22)
Uganda	68	56.2	55.7 (47.2, 65.7)	1
TB site				
Pulmonary	137	53.7	53.3 (38.5, 73.6)	1
Extrapulmonary	19	55.9	54.9 (48.8, 61.7)	1.03 (0.73, 1.45)
Patient type				
New	141	55.1	55.0 (48.9, 61.8)	1
Relapse	16	51.6	51.5 (36.2, 73.2)	0.94 (0.65, 1.35)
HIV status				
HIV-negative	82	58.2	58.3 (50.6, 67.2)	1
HIV-positive	76	50.3	51.0 (42.7, 60.8)	0.87 (0.70, 1.09)
Resided in area^4^ where health facility is located				
Yes	93	53.8	54.6 (48.4, 61.5)	1
No	15	60.0	54.6 (38.5, 77.5)	1.00 (0.68, 1.46)
Marital status				
Married/cohabitating with partner	81	54.4	54.4 (46.9, 63.0)	1
Single, widowed, divorced, or separated	77	53.8	55.0 (46.9, 64.5)	1.01 (0.82, 1.25)
Highest level of education completed				
Less than primary school	55	49.1	49.8 (41.4, 60.0)	1
Primary school	78	56.1	56.9 (49.2, 65.8)	1.14 (0.91, 1.43)
Form 6 or above	25	61.0	52.5 (29.8, 92.5)	1.19 (0.88, 1.62)
Employment status				
Formally employed	26	54.2	54.5 (42.3, 70.4)	0.99 (0.75, 1.32)
Informally employed	83	54.6	55.0 (47.6, 63.5)	1
Not employed, seeking work	22	52.4	52.9 (39.8, 70.3)	0.96 (0.70, 1.31)
Not employed, not seeking work	27	54.0	54.7 (41.9, 71.3)	0.99 (0.74, 1.33)
Worked in the fishing industry in the past 12 months				
No	136	55.1	55.8 (49.6, 62.6)	1
Yes	22	48.9	48.4 (34.9, 66.9)	0.87 (0.62, 1.22)
Worked in a mine in the past 12 months				
No	139	53.9	54.9 (48.9, 61.6)	1
Yes	17	53.1	52.9 (38.1, 73.3)	0.96 (0.69, 1.35)
Any household member(s) went to bed hungry in the past 30 days				
No	111	54.7	55.5 (48.9, 63.0)	1
Yes	42	50.6	52.3 (42.5, 64.4)	0.94 (0.74, 1.19)

^1^ Clinic presentation delay was defined as waiting more than 6 weeks after the onset of TB symptoms to seek care at a health facility.

^2^ Clinic presentation delay was assessed in face-to-face interviews of subcohort members. Prevalence was computed among n = 292 subcohort members with defined time to clinic presentation.

^3^ Prevalence and prevalence ratio estimates for the cohort and corresponding standard errors were computed in each of 200 imputed cohort data sets and summarized using Rubin’s rules.

^4^ Sub-county in Kenya; district in Tanzania and Uganda.

Data were collected at 12 health facilities in the Lake Victoria region of East Africa in the East Africa TB/HIV and Mobility Study (2019).

Among people who experienced clinic presentation delay, 64 (40.5%) said they did not present to a health facility sooner because they first tried other treatments. An additional 33 people (20.9%) indicated that they did not think their symptoms were caused by TB, due to limited knowledge of TB symptoms or their perception of their symptoms as non-severe. Thirty-five people (22.2%) said they did not know where to go for care. All other reasons were reported by less than 5% of respondents.

**Fig 2:**
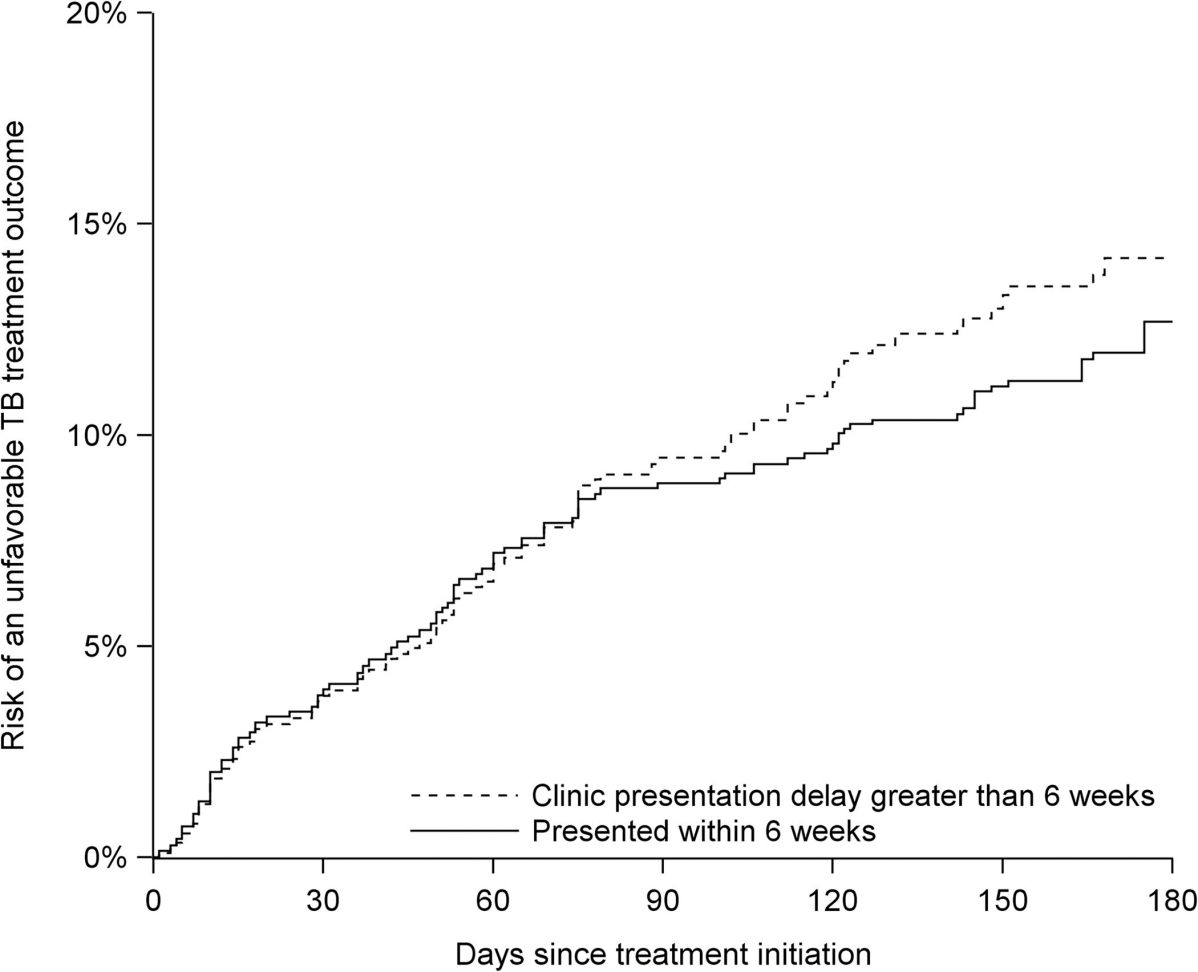
Risk of an unfavorable TB treatment outcome by clinic presentation delay. Risks were estimated from the time of TB treatment initiation up to 180 days, among people who did and did not experience clinic presentation delay. An unfavorable TB treatment outcome was defined as any outcome of: treatment failed, died, or lost to follow-up. Clinic presentation delay was defined as presenting to a health facility more than 6 weeks after the onset of TB symptoms. Data are from the East Africa TB/HIV and Mobility Study (2019).

Among cohort members with recorded treatment outcomes (n = 596, 76.9%), 103 had an unfavorable TB outcome: 64 died, 35 were lost to follow-up, and 4 experienced treatment failure. The full distribution of outcomes is presented in the S2 Table. The mean duration of follow-up in the risk period was 144 (standard deviation: 48.6) days. The 180-day risk of an unfavorable TB treatment outcome in the cohort overall was 13.6% (95% CI: 11.0%, 16.1%) (Table 3). The 180-day risk was 12.7% (95% CI: 5.1%, 20.3%) among those with clinic presentation delay, and 14.2% (95% CI: 8.0%, 20.4%) among those who presented to a clinic sooner (Fig 2), for a risk difference of 1.5 (95% CI: -8.3, 11.3) percentage points. The association between clinic presentation delay and 180-day risk of an unfavorable outcome was slightly stronger among those without (vs. with) TB-associated HIV, and among female (vs. male) cohort members; however, estimates were imprecise (Table 3). The HIV- and sex-stratified risks of an unfavorable TB treatment outcome over the 180-day period since TB treatment initiation among those who did and did not experience clinic presentation delay are presented in the S1 File.

**Table 3:** Risk of an unfavorable TB treatment outcome by clinic presentation delay. The 180-day risk of an unfavorable TB treatment outcome was estimated for the cohort overall and according to experience of clinic presentation delay. Results are also shown for strata defined by HIV status and sex. Risk differences present the associations between clinic presentation delay and an unfavorable TB treatment outcome at 180 days.

	180-day risk of an unfavorable TB treatment outcome^1^ (%) (95% CI)^2^	Risk difference (%) (95% CI)^3^
Overall	13.6 (11.0, 16.1)	
Presented within 6 weeks	12.7 (5.1, 20.3)	0
Clinic presentation delay greater than 6 weeks	14.2 (8.0, 20.4)	1.5 (-8.3, 11.3)
Among people without HIV	11.2 (7.7, 14.5)	
Presented within 6 weeks	9.9 (2.0, 17.9)	0
Clinic presentation delay greater than 6 weeks	12.0 (6.0, 18.1)	2.1 (-7.9, 12.1)
Among people with HIV	15.9 (12.0, 19.7)	
Presented within 6 weeks	14.9 (5.6, 24.3)	0
Clinic presentation delay greater than 6 weeks	16.6 (7.9, 25.3)	1.6 (-11.1, 14.4)
Among female cohort members	13.4 (9.4, 17.3)	
Presented within 6 weeks	12.2 (2.6, 21.8)	0
Clinic presentation delay greater than 6 weeks	14.2 (5.7, 22.7)	2.0 (-10.8, 14.8)
Among male cohort members	13.6 (10.2, 16.9)	
Presented within 6 weeks	12.9 (4.7, 21.0)	0
Clinic presentation delay greater than 6 weeks	14.1 (7.6, 20.7)	1.3 (-9.2, 11.8)

^1^ This composite outcome was defined as any outcome of: treatment failed, died, or lost to follow-up.

^2^ Risks and standard errors were estimated in each of 200 imputed cohort data sets using the Nelson-Aalen estimator, then summarized using Rubin’s rules.

^3^ Standard errors for risk differences were calculated using the delta method.

Data were collected at 12 health facilities in the Lake Victoria region of East Africa in the East Africa TB/HIV and Mobility Study (2019).

When restricting to cohort members with measured values for clinic presentation delay, we found that throughout the 180-day follow-up period, the risk of an unfavorable TB treatment outcome was higher among those who experienced clinic presentation (S2 File). When examining mortality as the outcome of interest, we estimated that the 180-day risk of death was 5.8 (95% CI: -2.1, 13.6) percentage points higher among cohort members who experienced clinic presentation delay as compared to those who presented to a health facility within 6 weeks of the onset of TB symptoms (S3 File).

## Discussion

In this study, we reported prevalence of and reasons for clinic presentation delay, explored factors associated with this delay, and estimated the association between clinic presentation delay and an unfavorable TB treatment outcome. We found that the risk of an unfavorable outcome was slightly higher among those who waited more than 6 weeks to seek care at a health facility, though the risk difference was not statistically significant. None of the assessed clinical or sociodemographic characteristics was strongly associated with clinic presentation delay.

We estimated that more than half (54.7%; 95% CI: 48.9%, 61.2%) of people initiating TB treatment at the selected health facilities experienced clinic presentation delay. This type of delay (elsewhere, often termed “patient delay”) has been described previously in some areas of East Africa, including Pwani region [23], Dar es Salaam [24], and Mwanza region [25], Tanzania. In this multi-county study, we estimated that the prevalence of clinic presentation delay was similar across cohort members in Kenya, Tanzania, and Uganda. This indicates the pervasive nature of clinic presentation delay throughout the region. Studies elsewhere in East Africa have found clinic presentation delay to be associated with sex [24, 26, 27], older age [25], lower educational attainment [25], marital status [28], unemployment [27], and health beliefs [23]; however, we did not identify any strong correlates of clinic presentation delay in this cohort.

In this study, we found a small association between clinic presentation delay and unfavorable TB treatment outcomes. This is compatible with a range of hypotheses, including the null. Other studies assessing the association between clinic presentation delay or total delay (i.e., time from TB symptoms to treatment initiation) and sub-optimal TB treatment outcomes have reported the same direction of effect as what we report here [29–36].

Assessment of clinic presentation delay was subject to interviewer and recall biases, which may have caused us to underestimate the prevalence of clinic presentation delay. We also assessed clinic presentation delay as a categorical rather than continuous variable in the subcohort survey. We are therefore unable to describe time to clinic presentation in further detail or consider alternative thresholds that could be relevant to TB treatment outcomes. In addition, because clinic presentation delay was assessed only in the subcohort, inferences in the full cohort depend on the validity of the process we used to impute missing clinic presentation delay data. Our results could be biased if we omitted important variables associated with both clinic presentation delay and an unfavorable outcome. We found, however, that the direction of the association between clinic presentation delay and an unfavorable TB treatment outcome was consistent whether including or excluding cohort members with an imputed value for clinic presentation delay. Some TB treatment outcomes may have been misclassified due to incomplete records, incomplete death ascertainment, or silent transfers. Furthermore, we used a composite outcome, which may obscure associations between clinic presentation delay and individual treatment outcomes. The composite outcome is operationally useful, however, given the desirability of reducing all component outcomes (death, loss to follow-up, and treatment failure), and given that similar interventions would likely be used to prevent each outcome. The direction of association between clinic presentation delay and mortality was consistent with the direction of association between clinic presentation delay and the composite outcome. The point estimate of the 180-day risk difference was larger when considering mortality as compared to the composite outcome; however, the risk estimates for mortality may be sensitive to incomplete death ascertainment, particularly among people lost to follow-up. Finally, our findings may not generalize to people in care at health facilities dissimilar to those selected for this study, such as at facilities that are not linked to Xpert MTB/RIF testing, or where the profile of clinic presentation delay is dissimilar.

The high prevalence and generalized nature of clinic presentation delay in communities near Lake Victoria suggests a need for multi-level community-based interventions that address known barriers to care. Such interventions may involve, for example, providing patient-centered services close to where people with TB live and work. Given that members of the subcohort commonly cited non-specificity and mildness of symptoms as reasons for clinic presentation delay, time to diagnosis and treatment may be improved by increasing public awareness of the range and potential mild presentation of early TB symptoms. About one in five subcohort members with clinic presentation delay reported that they were unsure where to go for care. Informational campaigns about where to seek care for TB symptoms could help to mitigate delays among people experiencing this barrier. Many (40.5%) of those with clinic presentation delay reported seeking traditional medicine or medications that were not specifically anti-TB drugs before they visited a healthcare facility. This has been previously described in the region [9, 26]. Increasing or introducing TB screening into these non-clinical settings may be effective in reducing clinic presentation delays. Still, the often low positive predictive value of symptom-based TB screening [37–40] warrants consideration. Especially in highly resource-limited settings, the costs and potential benefits of expanded TB screening should be weighed against those of other efforts to improve individual and public health [41].

This study demonstrates an efficient method to study yet-unmeasured factors in a defined population by collecting new data among a subset of a population while leveraging extant data to produce estimates generalizable to the broader population. This approach can be especially useful in healthcare settings, where routine medical records exist and results can inform interventions among an accessible population (i.e., people in care). Substantively, our results provide important insights into clinic presentation delays occurring in communities near Lake Victoria and into the relationship between clinic presentation delay and unfavorable TB treatment outcomes.

## Supporting information

S1 Checklist Inclusivity in global research checklist. (DOCX)

S1 Table Variables used in multiple imputation. (DOCX)

S2 Table TB treatment outcomes in the full study cohort. (DOCX)

S1 File Risk of an unfavorable TB treatment outcome by clinic presentation delay, stratified by HIV status and sex. (DOCX)

S2 File Risk of an unfavorable TB treatment outcome by clinic presentation delay, restricting to cohort members with measured values for clinic presentation delay. (DOCX)

S3 File Risk of death by clinic presentation delay. (DOCX)
