# Supplementary material for: Clinic presentation delay and tuberculosis treatment outcomes in the Lake Victoria region of East Africa: A multi-site prospective cohort study
Source: PLOS Glob Public Health. 2023 Aug 30;3(8):e0002259. doi: 10.1371/journal.pgph.0002259 (PMC10468066; doi:10.1371/journal.pgph.0002259)
Supplement: S1 Table — (DOCX) [file pgph.0002259.s002.docx]

**S1 Table.** **Variables used in multiple imputation.**

| **Variable** | **Type** | **Coding for imputation models** | **Missing values?** | **Method for imputation** |  |
| --- | --- | --- | --- | --- | --- |
| Sex | Binary | Female versus Male | No | N/A |  |
| Age | Continuous | Linear predictor and 3 restricted quadratic spline basis functions | No | N/A |  |
| HIV status | Binary | HIV-positive versus HIV-negative | No | N/A |  |
| Country where initiating TB treatment | Nominal | Kenya, Tanzania, or Uganda | No | N/A |  |
| Patient type | Binary | New versus Relapse or return after treatment failure or loss to follow-up | Yes | Discriminant function |  |
| TB site | Binary | Pulmonary versus Extrapulmonary | Yes | Discriminant function |  |
| Partnered | Binary | Married or cohabitating with a sexual partner versus Single, divorced, separated, or widowed | Yes | Discriminant function |  |
| Household hunger | Binary | Any member of the household went to bed hungry in the past 30 days versus No household members went to bed hungry in the past 30 days | Yes | Discriminant function |  |
| Employment status | Nominal | Formally employed, informally employed, not employed and seeking work, or not employed and not seeking work | Yes | Discriminant function |  |
| Educational attainment | Ordinal | Less than primary school; Primary school; Form 6; or College, vocational or tertiary school | Yes | Logistic |  |
| Recent work in the fishing industry | Binary | Worked in the fishing industry in the past 12 months, versus Did not work in this industry in the past 12 months (including those who did not work at all in the past 12 months) | Yes | Discriminant function |  |
| Recent work in the mining industry | Binary | Worked in the mining industry in the past 12 months, versus Did not work in this industry in the past 12 months (including those who did not work at all in the past 12 months) | Yes | Discriminant function |  |
| Resided near health facility | Binary | Resided in the same district (if in Uganda or Tanzania) or subcounty (if in Kenya) as the health facility where enrolled in TB treatment versus Resided further away | Yes | Discriminant function |  |
| Subcohort membership | Binary | In subcohort, versus not in subcohort | No | N/A |  |
| Outcome indicator variable | Nominal | Experienced an unfavorable TB treatment outcome, experienced a competing event, or was censored at time *t* | No | N/A |  |
| Cumulative baseline hazard of unfavorable TB treatment outcome | Continuous | Approximated by the Nelson–Aalen estimator of the hazard at time *t ^1^* | No | N/A |  |
| **^1^** White IR, Royston P. Imputing missing covariate values for the Cox model. *Stat Med* 2009; 28:1982–1998. | | | | | |
