# Supplementary material for: Clinic presentation delay and tuberculosis treatment outcomes in the Lake Victoria region of East Africa: A multi-site prospective cohort study
Source: PLOS Glob Public Health. 2023 Aug 30;3(8):e0002259. doi: 10.1371/journal.pgph.0002259 (PMC10468066; doi:10.1371/journal.pgph.0002259)
Supplement: S2 Table — (DOCX) [file pgph.0002259.s003.docx]

**S2 Table. TB treatment outcomes in the full study cohort.**

| **Individual TB treatment outcome** | **n** | **%** |
| --- | --- | --- |
| Cured | 88 | 11.4 |
| Treatment completed | 228 | 29.4 |
| Treatment failed | 4 | 0.5 |
| Died | 64 | 8.3 |
| Lost to follow-up | 35 | 4.5 |
| Not evaluated (transferred out or unknown) ^1^ | 151 | 19.5 |
| Transferred out ^2^ | 17 | 2.2 |
| Treatment stopped due to other medical condition | 1 | 0.1 |
| Still on treatment: Confirmed via tracing survey | 50 | 6.5 |
| Presumed still on treatment: On treatment for less than 6 months and no outcome recorded | 122 | 15.7 |
| No outcome recorded 6 or more months since treatment initiation | 15 | 1.9 |
| **Composite outcome:** Unfavorable TB treatment outcome ^3^ | 103 | 13.3 |
| ^1^ Outcome category used in TB treatment registers in Kenya and Tanzania.  ^2^ Outcome category used in TB treatment registers in Uganda.  ^3^ Composite outcome: includes outcomes of treatment failed, died, and lost to follow-up.  Data were collected at 12 health facilities in the Lake Victoria region of East Africa in the East Africa TB/HIV and Mobility Study (2019). | | |
