# Supplementary material for: Clinic presentation delay and tuberculosis treatment outcomes in the Lake Victoria region of East Africa: A multi-site prospective cohort study
Source: PLOS Glob Public Health. 2023 Aug 30;3(8):e0002259. doi: 10.1371/journal.pgph.0002259 (PMC10468066; doi:10.1371/journal.pgph.0002259)
Supplement: S1 File — (DOCX) [file pgph.0002259.s004.docx]

**S1 File. Risk of an unfavorable TB treatment outcome by clinic presentation delay, stratified by HIV status and sex.**

*Strata defined by HIV status*

S1 File Fig 1 presents, for strata of cohort members defined by HIV status, the risk of unfavorable TB treatment outcome over 180 days since TB treatment initiation among those who did and did not experience clinic presentation delay.


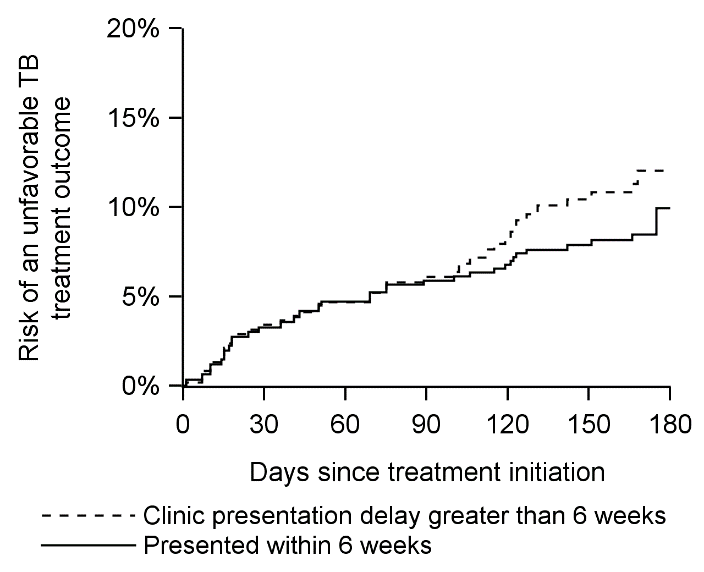


**a.**


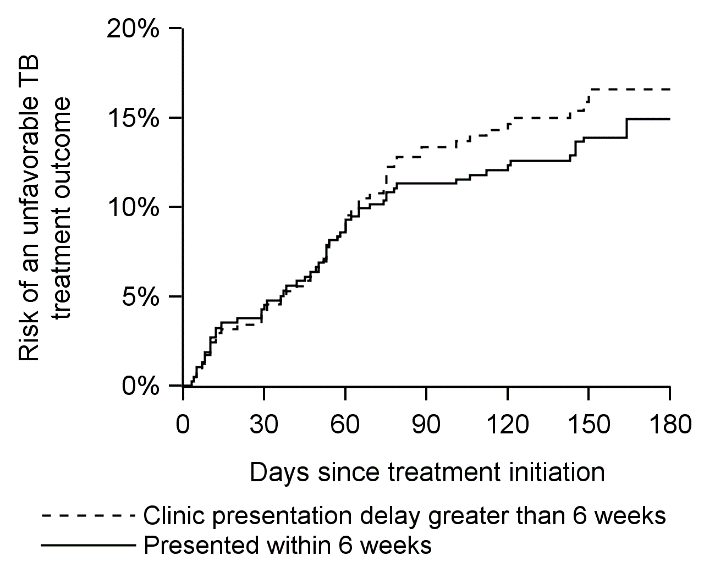


**b.**

**S1 File Fig 1.** **Risk of an unfavorable TB treatment outcome across 180 days since TB treatment initiation, among people (a) without HIV and (b) with HIV, by experience of clinic presentation delay.** Data are from the East Africa TB/HIV and Mobility Study (2019).

*Strata defined by sex*

S1 File Fig 2 presents, for strata of cohort members defined by sex recorded in clinical records, the risk of unfavorable TB treatment outcome over 180 days since TB treatment initiation among those who did and did not experience clinic presentation delay. For female cohort members who experienced clinic presentation delay, supremum risks are presented due to non-monotonicity of the risk function at two time points (t = 128 and t = 144).


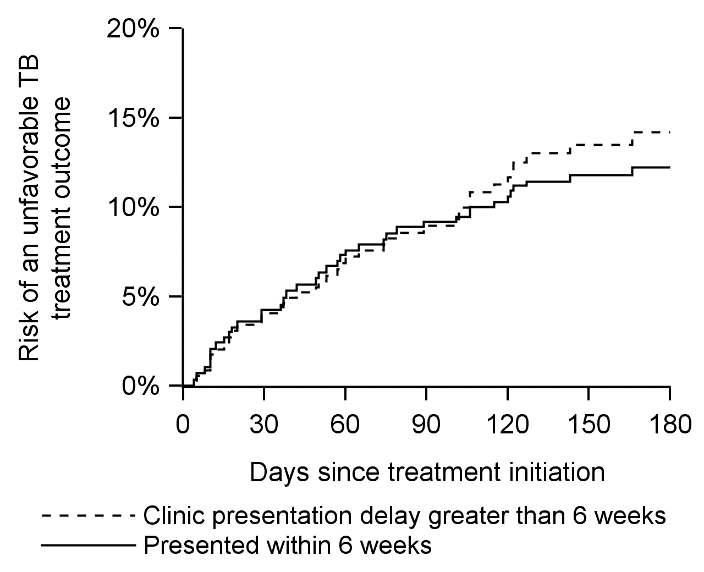


**a.**


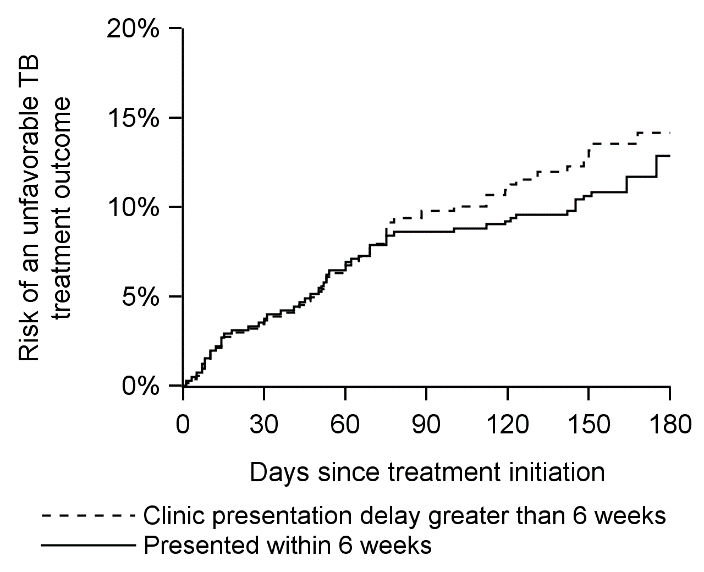


**b.**

**S1 File Fig 2. Risks of unfavorable TB treatment outcome over 180 days since TB treatment initiation, among (a) female cohort members, and (b) male cohort members, by experience of clinic presentation delay.** Data are from the East Africa TB/HIV and Mobility Study (2019).
