# Supplementary material for: Clinic presentation delay and tuberculosis treatment outcomes in the Lake Victoria region of East Africa: A multi-site prospective cohort study
Source: PLOS Glob Public Health. 2023 Aug 30;3(8):e0002259. doi: 10.1371/journal.pgph.0002259 (PMC10468066; doi:10.1371/journal.pgph.0002259)
Supplement: S2 File — (DOCX) [file pgph.0002259.s005.docx]

**S2 File. Risk of an unfavorable TB treatment outcome by clinic presentation delay, restricting to cohort members with measured values for clinic presentation delay.**

The figure below (Fig) presents risks of an unfavorable TB treatment outcome over 180 days since TB treatment initiation when the analytic data set is restricted to cohort members with measured (not imputed) values for clinic presentation delay (n = 292). Data were weighted in this complete case analysis to account for differences in the probability of having a defined time to clinic presentation. This probability differed among subcohort members given the use of health facility-specific recruitment targets to recruit cohort members for the subcohort survey.

The marginal risk of an unfavorable TB treatment outcome is lower in the subcohort than in the cohort overall, as only 12 unfavorable outcomes were recorded among members of the subcohort.


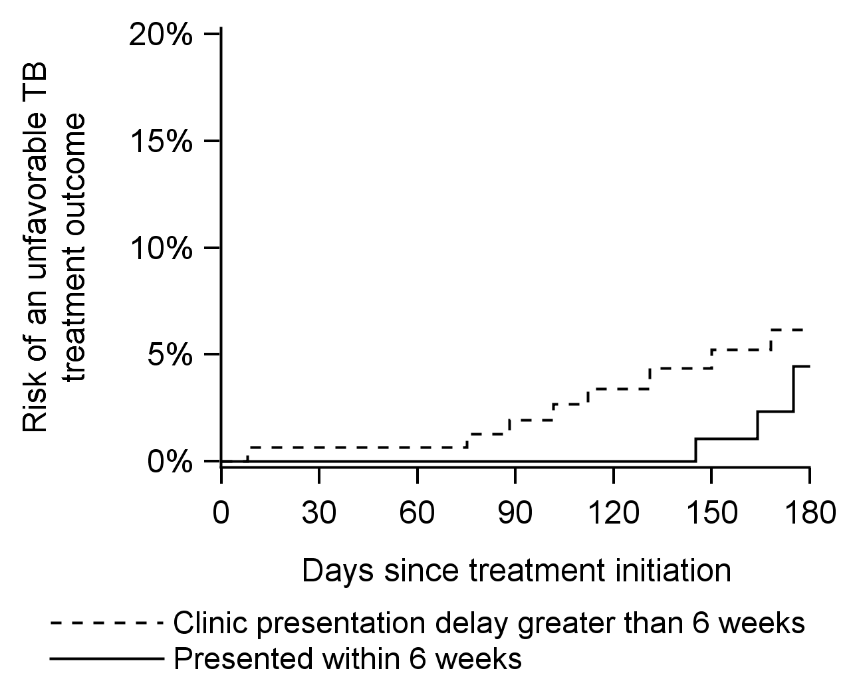


**Fig.** **Risk of an unfavorable TB treatment among cohort members with measured values for clinic presentation delay.** Data from cohort members with defined values for clinic presentation delay (n = 292) were weighted to account for differences in the probability of recruitment for the subcohort survey due to health facility and HIV status. Risks were estimated from the time of TB treatment initiation up to 180 days among people who did and did not experience clinic presentation delay. Clinic presentation delay was defined as presenting to a health facility more than 6 weeks after the onset of TB symptoms. Data are from the East Africa TB/HIV and Mobility Study (2019).
