# Supplementary material for: Clinic presentation delay and tuberculosis treatment outcomes in the Lake Victoria region of East Africa: A multi-site prospective cohort study
Source: PLOS Glob Public Health. 2023 Aug 30;3(8):e0002259. doi: 10.1371/journal.pgph.0002259 (PMC10468066; doi:10.1371/journal.pgph.0002259)
Supplement: S3 File — (DOCX) [file pgph.0002259.s006.docx]

**S3 File. Risk of death by clinic presentation delay.**

We repeated the multiple imputation process, using an imputation model that included death as the outcome to ensure congeniality between the imputation and analysis models. Because follow-up did not extend beyond the first recorded TB treatment outcome, we treated outcomes of treatment failure and loss to follow-up as censoring events in this analysis. The results, presented in the following Fig and Table, may be sensitive to misclassification among the component outcomes included in the composite outcome. In particular, some proportion of deaths may have been recorded in the TB registers as losses to follow-up if death ascertainment was incomplete.

**
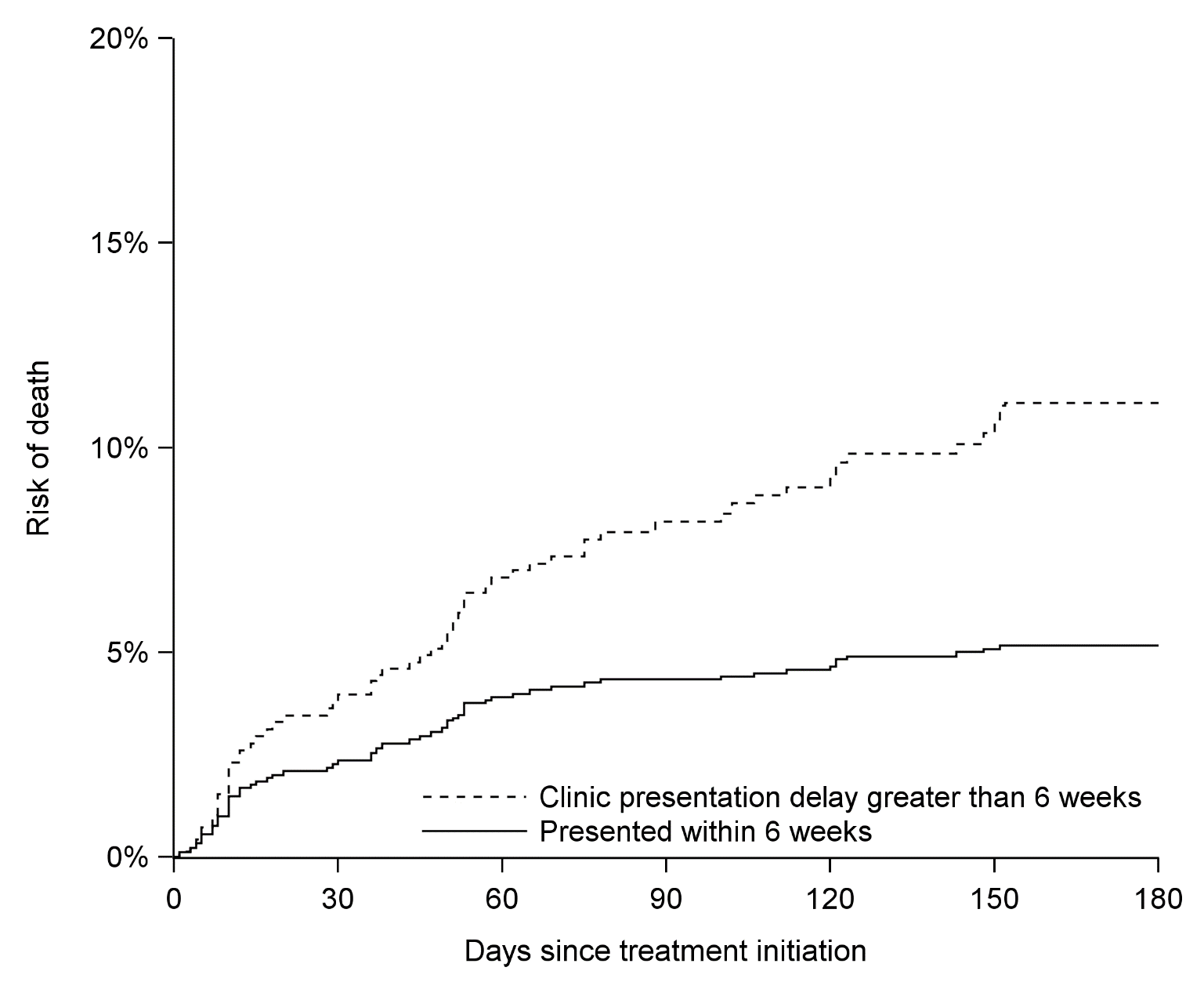
**

**Fig. Risk of death by clinic presentation delay.** Risks were estimated from the time of TB treatment initiation up to 180 days, among people who did and did not experience clinic presentation delay. Clinic presentation delay was defined as presenting to a health facility more than 6 weeks after the onset of TB symptoms. Data are from the East Africa TB/HIV and Mobility Study (2019).

**Table.****Risk of death by clinic presentation delay.** The 180-day risk of death was estimated for the cohort overall and according to experience of clinic presentation delay. Risk differences present the association of clinic presentation delay and mortality at 180 days.

|  | 180-day risk of specified outcome (%) (95% CI)^1^ | Risk difference (%) (95% CI)^2^ |
| --- | --- | --- |
| Overall | 8.4 (6.4, 10.5) |  |
| Presented within 6 weeks | 5.2 (0.0, 11.0) | 0 |
| Clinic presentation delay greater than 6 weeks | 10.9 (5.7, 16.2) | 5.8 (-2.1, 13.6) |
| ^1^ Risks and standard errors were estimated in each of 200 imputed cohort data sets using the Nelson-Aalen estimator, then summarized using Rubin’s rules.  ^2^ Standard errors for risk differences were calculated using the delta method.  Data were collected at 12 health facilities in the Lake Victoria region of East Africa in the East Africa TB/HIV and Mobility Study (2019). | | |
